# Time of ureteral ejection of sodium fluorescein in the cystoscopic assessment of ureteral patency in patients undergoing total laparoscopic hysterectomy

**DOI:** 10.4274/jtgga.galenos.2019.2019.0091

**Published:** 2020-03-06

**Authors:** Fred Morgan-Ortiz, Fred Valentín Morgan-Ruiz, Josefina Báez-Barraza, José Cándido Ortiz-Bojórquez, Jesús Israel Martínez-Félix, Felipe de Jesús Peraza-Garay

**Affiliations:** 1 Department of Obstetric and Gynecology, Universidad Autonoma de Sinaloa, Sinaloa, Mexico

**Keywords:** Sodium fluorescein, ureteral patency, ejection time, laparoscopic hysterectomy

## Abstract

**Objective:**

To evaluate the time of ureteral ejection of intravenous sodium fluorescein in the assessment of ureteral patency in patients undergoing total laparoscopic hysterectomy (TLH).

**Material and Methods:**

Fifty-four women undergoing TLH were studied in a public teaching hospital in Culiacan, Sinaloa, Mexico. They underwent cystoscopic evaluation of ureteral patency after intravenous administration of 100 mg of sodium fluorescein. The present study analyzed the time elapsed in minutes from the intravenous administration of fluorescein to the outflow of stained urine by one or both ureteral meatus, the degree of urine staining, and the impact of body mass index (BMI) (BMI; normal, overweight, and obesity) on ejection time.

**Results:**

The overall average time elapsed to visualize the ejection of fluorescein through at least one ureteral meatus was 7.5 minutes [95% confidence interval (CI): 6.3-8.7]. There were no significant differences in the time of ureteral ejection of fluorescein taking BMI into account (p=0.579), with a mean time for normal BMI of 8.1 minutes (95% CI: 5.1-11.2), for overweight of 7.0 minutes (95% CI: 5.5-8.5), and for obesity of 7.8 minutes (95% CI: 5.3-10.3).

**Conclusion:**

Intravenously administered 10% sodium fluorescein dye is rapidly eliminated and strongly stains urine, which makes it useful for identifying ureteral patency during cystoscopy after TLH. Fluorescein excretion is not affected by patient BMI.

## Introduction

After cesarean section, hysterectomy is the surgical procedure most commonly performed for benign indications. In the United States (US), approximately 600,000 hysterectomies are performed per year using one of three approaches: abdominal, vaginal, and laparoscopic (1).

Although laparoscopic hysterectomy (LH) has many advantages over laparotomic hysterectomy, it also has disadvantages, and there is an increased risk of complications when surgeons with little experience perform the procedure. Urinary tract injuries (bladder and ureter) are among the most common complications associated with LH (2,3,4). The frequency of urinary tract injuries reported for total laparoscopic hysterectomy (TLH) (0.31%) is roughly similar to that reported for laparotomic hysterectomy (0.03-2.0%), with hematuria as the main sign of injury (5,6,7).

Although the risk of injuring both the bladder and ureter can be high during TLH, most injuries can be identified by cystoscopy (8).

Gynecologic surgery causes 75% of iatrogenic injuries to the bladder and ureter. Visual inspection alone will miss many of these injuries. Furthermore, visual evaluation of ureteral peristalsis during the procedures is not reliable. Less than 50% and 25% of cases of ureteral and bladder injuries respectively are detected by visual inspection when intraoperative cystoscopy is not performed (8).

Due to this low rate of detection of ureteral injuries during gynecological surgery, the American College of Obstetricians and Gynecologists recommends that all gynecological surgeons should perform diagnostic cystoscopies for optimal patient care, with the aim of evaluating bladder and ureteral integrity (9).

When performing cystoscopy, it is advisable to use dyes to better evaluate ureteral integrity. For this purpose, many stains have been used, including indigo carmine, methylene blue, 10% sodium fluorescein, phenazopyridine, and vitamin B12, among others (10,11,12,13,14,15,16). Several studies have shown that the use of indigo carmine during cystoscopy is useful for detecting ureteral injuries, but since 2014, this drug has not been marketed in the US (10,11,12). Methylene blue in solution for intravenous use at a dose of 50 mg (5 mL of a 10 mg/mL solution) is mainly eliminated in urine, which stains blue and is easily visible during cystoscopy. One disadvantage of methylene blue is that it interferes with pulse oximetry by altering oxygen saturation readings and can result in a serotonergic syndrome when administered concomitantly with serotonin reuptake inhibitors or monoamine oxidase inhibitors (13). Another intravenous agent that promises to be useful to stain urine during diagnostic cystoscopy after a gynecological procedure and that has been used extensively in ophthalmology is 10% sodium fluorescein. It is fast acting and well tolerated, but there are few reports on its use in gynecological procedures. It can be used intravenously at doses of 0.25 to 1 mL (25 to 100 mg) and is rapidly eliminated in urine, giving urine a bright yellow color easily visible during ureteral emptying (14).

The purpose of the present study was to evaluate the use of intravenous sodium fluorescein in the cystoscopic assessment of bladder and ureteral integrity and to determine the time of ureteral ejection in patients undergoing TLH.

## Material and Methods

After approval from the Local Research and Ethics Committee of the Civil Hospital of Culiacan, Sinaloa, Mexico (decision no: 306), and after obtaining the written informed consent of the patients, a prospective, descriptive, and observational study was conducted in 54 healthy patients submitted for TLH, who underwent cystoscopic evaluation of bladder and ureteral integrity after intravenous administration of 100 mg of sodium fluorescein diluted in 10 cc of saline solution (1 mL of 10% sodium fluorescein containing 0.1 g of fluorescein; Alcon Laboratories Inc., Fort Worth, TX). Fluorescein was administered after laparoscopic port closure. All patients were asked about history of allergic reactions before starting the surgical procedure.

Cystoscopy was conducted with the patient in the lithotomy position, under general anesthesia, and after injection of 200 mL of saline solution through the bladder catheter. After bladder distention with saline solution, a 5 mm lens connected to an endoscopic camera was introduced to evaluate bladder integrity, identify the ureteral meatus, and visualize the ejection of urine through both meatus.

**Figure 1:**
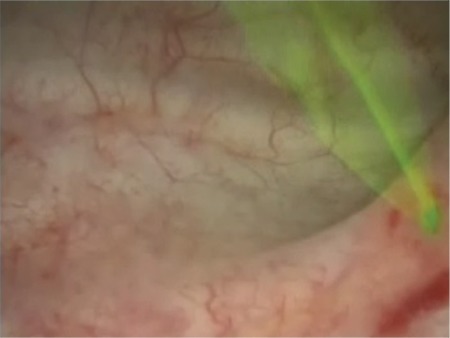
Observation by cystoscopy of the ejection of fluorescein through the left ureteral meatus

The primary variable was the time elapsed from the administration of fluorescein to the cystoscopic observation of the outflow of dye-stained urine through at least one ureteral meatus (Figure 1). Secondary variables were patient age, body mass index (BMI), surgical time, rate of bladder and ureteral injuries identified during cystoscopy, frequency of hematuria and rate of adverse events associated with the drugs including nausea, vomiting, headache, gastrointestinal conditions, syncope, hypotension, severe shock, seizures, thrombophlebitis at the injection site and other symptoms and signs of hypersensitivity. Adverse events and urinary complications were evaluated for at least four weeks after the surgical procedure. To analyze if the time of ureteral ejection of fluorescein could be affected by selected variables, it was compared in relation to BMI (normal, overweight, and obesity) and the surgical time of the procedure.

### Statistical analysis

To compare time of ejection of fluorescein between the BMI groups, the log-rank test was used with 95% confidence intervals. A p-value <0.05 was considered statistically significant. The data were analyzed with SPSS v24 (IBM Inc., Armonk, NY, USA) statistical software.

## Results

Fifty-four patients with a mean age of 45.3 years [standard deviation (SD) ±5.5] and mean BMI of 28.8 kg/m2 (SD ±4.5) were included. The number of patients with normal BMI was 12 (22.2%), with overweight 25 (46.3%), and with obesity was 17 (31.5%). Mean parity was 2.9 (SD ±1.3); the number of patients with at least one previous cesarean section was 38 (70.4%), of which 22 (57.8%) had a history of three cesarean sections. The main indication for hysterectomy was abnormal uterine bleeding (96.3%) (Table 1). All patients with abnormal uterine bleeding were given medical treatment before performing LH.

Mean surgical time for hysterectomy was 104.2 minutes (SD ±37.5), mean operative bleeding was 114.4 mL (SD ±37.5), and mean uterine weight was 262.3 g (SD ±167.2).

**Figure 2:**
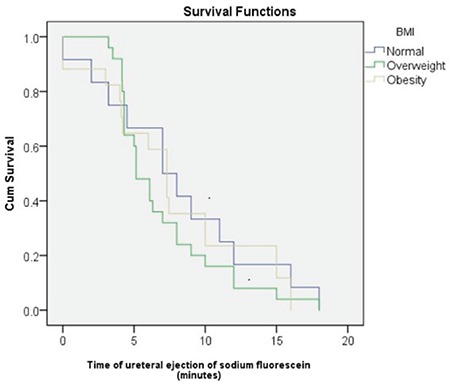
Survival curve showing the time of ureteral ejection of fluorescein by body mass index groups BMI: Body mass index

There were no significant differences in the time to ureteral ejection of fluorescein between the BMI groups (log-rank=1.093, p=0.579). Mean overall time of ureteral ejection was 7.5 min [95% confidence interval (CI): 6.3-8.7] (Table 2). By BMI group, the mean time for the normal BMI group was 8.1 min (95% CI: 5.1-11.2), for the overweight group was 7.0 minimum (95% CI: 5.5-8.5), and for the obese group was 7.8 min (95% CI: 5.3-10.3), (p=0.560) (Table 2, Figure 2).

There was only one bladder injury in a patient with a previous cesarean section repaired laparoscopically in two planes with vicryl 000 suture; the integrity of the suture line was previously corroborated with the retrograde instillation of 200 mL of physiological solution plus 1 mL of 10% sodium fluorescein. No ureteral injuries were observed during the study period. There were no adverse effects after the administration of fluorescein.

## Discussion

Lower urinary tract injuries are serious potential complications related to TLH due to the anatomical closeness between the bladder, ureter, and uterus. A recent study on LH, in which the ureter was identified prior to the procedure, reported a frequency of 0.31% in lower urinary tract injuries, which included six bladder injuries and four ureter injuries (4). In this series of 54 patients, there were no ureteral injuries, and there was only one case of bladder injury in a patient with bladder adhesions from previous cesarean sections. This complication rate is similar to that reported for laparotomy hysterectomy (0.03-2.0%) in other studies (5,6).

Hematuria is one of the signs that may suggest lower urinary tract injury, mainly bladder injury, but not of the ureter, as the latter may be asymptomatic and manifest late. In one study, the frequency of hematuria was 2.1%, and that of lower urinary tract injury was 1.6% (7). In the present study, 26 patients had hematuria, and there were no cases of ureteral injury. Therefore, the results of the present study suggest that hematuria is not a good indicator of bladder or ureteral injury.

The most widely used stains to evaluate bladder and ureteral integrity during intraoperative cystoscopy in gynecological procedures are indigo carmine and methylene blue, which are scarce in Mexico. Therefore, it was necessary to evaluate other available alternatives to determine if they are effective for staining urine and determining ureteral patency.

A dye that has been evaluated for urine staining is 10% sodium fluorescein (widely used as a fluorescent tracer in many fields, mainly in retinal angiography), which is easily seen during cystoscopic evaluation, but there are no reports on the average time of ureteral ejection in patients undergoing TLH (17,18). Doses ranging from 0.5 to 1.0 cc of 10% sodium fluorescein result in good visualization of ureteral jets (14).

Fluorescein is a hydroxyxanthene dye with a fluorescence motif-unlike most organic dyes and has been used in ophthalmology to demarcate retinal, choroidal, and iris vessels and help detect retinal and vascular abnormalities. It is administered intravenously at an adult dose of 500 mg/5 mL of 10% sodium fluorescein; the dye may take 10 to 15 seconds to appear in the choroidal and retinal vessels, although this may vary depending on the site, rapidity of the injection, and state of the systemic circulation (19). In ophthalmology, the doses of sodium fluorescein used for intravenous angiography are generally higher than those used in the present study, in which lower doses were used (500 mg vs 100 mg) (19).

Fluorescein and its metabolites are actively excreted by the kidneys and they can be detected in the urine after the first circulatory pass with excretion being completed within six to 12 hours after injection, although urinary fluorescence can be detected up to 36 hours after administration in patients with normal renal function (19).

In the present study, the time of ureteral ejection of 10% sodium fluorescein in the urine was not affected by the BMI of the patients, as no significant differences were found between patients with normal weight, overweight, and obesity. Nevertheless, the mean time of ureteral ejection of 10% sodium fluorescein tended to be higher in patients with normal weight compared to patients with overweight and obesity, which may be due to the greater impact of the pneumoperitoneum on plasma and renal flow in normal-weight or thin patients. However, without management to force diuresis in patients undergoing TLH, the times of ureteral ejection of fluorescein were variable, which may also reflect patients responding differently to physiological changes induced by the pneumoperitoneum. It is notable that in all of the patients in this series of cases, the pressure was maintained at 14 mmHg during the entire surgical procedure.

Sodium fluorescein is not free of adverse effects with headache, nausea, vomiting, hypotension, and anaphylaxis being the most common side effects reported (18). Anaphylaxis is a rare event, reported with a frequency of 0.083% in patients undergoing intravenous angiography. However, physicians who use intravenous sodium fluorescein should be aware of this complication and be prepared to manage it (19). In a series of 12 patients in which 1 mL (100 mg) of 10% sodium fluorescein was used, one patient experienced a transient yellowing of the palms and sclera (14). This differs from the results of this series of 54 patients undergoing LH, in which no adverse events occurred. This may be due to the use of very low dose intravenous sodium fluorescein compared with the doses used in retinal angiography (19).

Ten percent sodium fluorescein (dye) administered intravenously is rapidly eliminated and strongly stains urine with a yellowish-green color, making urine outflow easily visible on its ejection through the ureteral meatus during cystoscopy after TLH. As BMI does not interfere with the time of ureteral ejection of the stained urine during cystoscopy, it can be used in patients undergoing TLH to evaluate ureteral patency and bladder injuries.

## Figures and Tables

**Table 1:**
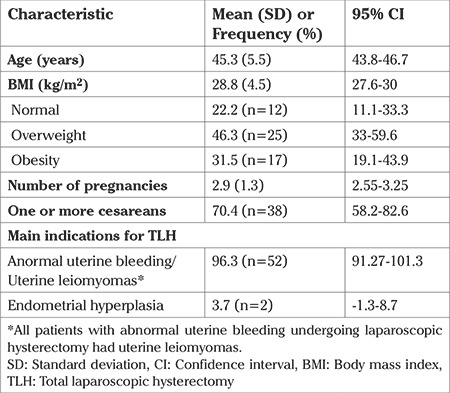
Demographic and clinical characteristics of the studied population

**Table 2:**
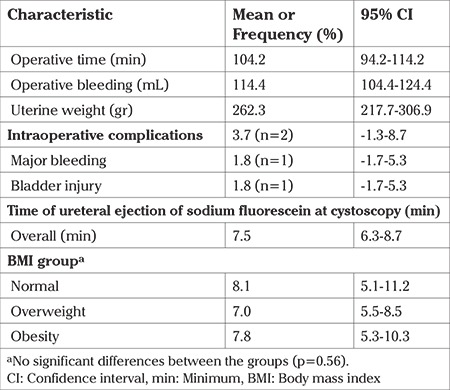
Operative characteristics and time of ureteral ejection of sodium fluorescein during cystoscopy of the studied population
